# Drs. W. C. Phelps and Alvin A. Hubbell

**Published:** 1911-11

**Authors:** 


The Erie County Hospital staff, at its regular meeting, September 18, 1911, passed memorial resolutions on the deaths of Drs. W. C. Phelps and Alvin A. Hubbell.

